# Changes in Dento-Facial Morphology Induced by Wind Instruments, in Professional Musicians and Physical Exercises That Can Prevent or Improve Them—A Systematic Review

**DOI:** 10.3390/life13071528

**Published:** 2023-07-08

**Authors:** Georgiana Macovei, Raluca Minea, Iarina Teodora Dumitraș, Cosmin Andrei Precup, Liliana Baroiu, Alexandru Nechifor, Adina Oana Armencia, Ana Cristina Lese

**Affiliations:** 1 Department of Oral and Dental Diagnostics, “Gr. T. Popa” University of Medicine and Pharmacy Iaşi, No. 16, Universității Street, 700115 Iași, Romania; dr_geo_m@yahoo.com (G.M.); iarina.dt@gmail.com (I.T.D.); oanaarmencia@yahoo.com (A.O.A.); 2 Department of Art History, Artistic Anatomy, Faculty of Visual Arts and Design, “George Enescu” National University of the Arts Iaşi, No. 189, Sărărie Street, 700451 Iaşi, Romania; 3 Department of Balneology, Medical Recovery and Rheumatology, “V.Babeş”University of Medicine and Pharmacy Timişoara, No. 2, Piața EftimieMurgu Street, 300041 Timișoara, Romania; precup.cosmin2@gmail.com; 4 Clinical Medical Department, Faculty of Medicine and Pharmacy,” Dunărea de Jos” University of Galați, No. 47, Domneasca Street, 800008 Galaţi, Romania; lilibaroiu@yahoo.com or liliana.baroiu@ugal.ro (L.B.); alexandrunechiformed@yahoo.com (A.N.); 5 Infectious Diseases Department, ”Sf. Cuv. Parascheva” Clinical Hospital of Infectious Diseases Galaţi, No. 393, Traian Street, 800179 Galaţi, Romania; 6 Multidisciplinary Integrated Center of Dermatological Interface Research Center (MICDIR), ”Dunărea de Jos” University of Galați, No. 47, Domneasca Street, 800008 Galaţi, Romania; 7 Department of Design, Faculty of Visual Arts and Design, “George Enescu” National University of the Arts Iaşi, No. 189, Sărărie Street, 700451 Iaşi, Romania; analese2000@yahoo.com

**Keywords:** wind instruments, dento-facial morphology, oral cavity, face muscles, breathing exercises

## Abstract

The aim of this study is to highlight the changes that occur regarding dento-facial morphology, facial, and masticatory muscles in musicians who play wind instruments. Anatomical and functional changes may occur after the long-term use of each type of wind instrument. We considered studies of the impact of playing the wind instrument on the masticatory muscle activity and the resulting modifications. Both children and adults can be affected by playing wind instruments in regard to tooth positioning and facial morphology. These changes relate to the type of wind instruments, the type of vowels and tones used by instrumentalists. There most valuable breathing techniques have been identified that improve the redistribution of pressure, with a minimizing effect on the morphological changes in the oral cavity and cephalic extremity, implicitly on the masticatory functional disorders. In addition to these beneficial effects on the stomatognathic system, these breathing techniques favorably increase lung capacity. Furthermore, a series of toning exercises for neck muscles—which are actively involved and overworked by wind instrument-playing artists—was identified. The study concludes that less experienced instrumentalists demonstrate increased facial muscle engagement, possibly leading to excessive strain, while experienced instrumentalists exhibit more optimized patterns of muscle activity. The novelty of this research lies in its interdisciplinary approach to understanding the influence of wind instruments on dento-facial morphology, addressing preventive and corrective measures to mitigate undesirable outcomes.

## 1. Introduction

If we draw a parallel between music and medicine, both of them date back to ancient times, with music considered as one of the arts with a significant impact on humans, and medicine regarded as the art of healing from the Latin “arsmedica”. For listeners, music has been shown to reduce stress, enhance neuroplasticity, and improve concentration, memory, creativity, and productivity. However, for artists, delivering a quality performance requires extensive practice and sustained study, especially in the field of music, where wind instrument players are particularly impacted. Recent studies have examined the anatomical and functional implications of these activities for optimal performance, with a focus on oro-facial changes in wind instrument players [1,2,3]. These changes may affect dental positioning, dental occlusion, oro-facial muscles, the lips, and the periodontium, with an interrelationship between the instrument and embouchure type, dental arches, oro-facial muscles, and lips. The impact of these changes is more significant, especially when the activity takes place during the growth period. Performing preventive and corrective exercises can assist wind instrument players in avoiding the detrimental effects of the forces exerted during playing [4,5]. Since the study of an instrument usually begins at a young age, it is crucial for parents and medical professionals who oversee the musician to be aware of these implications in order to provide personalized medical and physical guidance according to the artist’s needs for a future career with minimal damaging effects.

## 2. Materials and Methods

**Figure 1:**
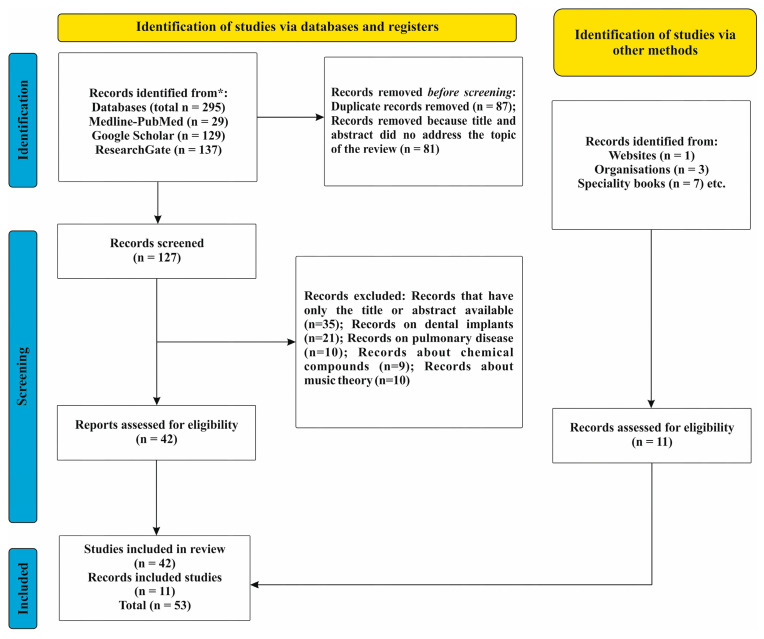
Prisma diagram. * PRISMA DIAGRAM FOR STUDY GROUP.

The research and extraction of eligible studies in this systematic review were conducted according to the guidelines outlined in the Preferred Reporting Items for Systematic Reviews and Meta-Analyses (PRISMA) statement (Figure 1) [6].

Search strategy: The search strategy employed multiple databases, including Medline-PubMed, Google Scholar, and Research Gate, to ensure comprehensive coverage of relevant studies. We utilized various combinations of the following keywords: “muscles of mimics”, “masticatory muscles wind instrument players”, “physical exercises for breathing”, “dental changes”, “class of wind instruments”, “trombones”, “clarinets”, “basses”, and “saxophones”. Due to the scarcity of articles addressing the specific topic of interest, a time frame for publication dates and restrictions on article type were not imposed. Furthermore, the reference lists of identified articles were manually reviewed to identify additional relevant resources. Recognizing the significance of this article, we also reviewed the resources of the reviewed articles.

Selection criteria: Two researchers independently reviewed all titles and abstracts from the medical field, while another two researchers did the same for the sports field, and an additional two researchers focused on the arts field. Any disagreements were resolved through consensus. The inclusion criteria for the study were as follows: (1) full-text articles and (2) studies regarding wind instrument players. The exclusion criteria consisted of: (1) incomplete articles with insufficient data; (2) articles related to instruments other than wind instruments; and (3) articles concerning occasional wind instrument players.

Data extraction: This was conducted by two independent reviewers using a standardized extraction table. This table included information such as the year of publication, country, first author, subject age, specific wind instrument played, dental changes, breathing exercises, and muscles involved. Discrepancies were resolved through discussion.

## 3. Results

A total of 295 articles were initially identified as follows: 29 records from Med-line-Pub Med, 129 from Google Scholar, and 137 from Research Gate. Of these, 168 were removed before screening as among them, 87 were duplicate records, and 81 were removed because the title and abstract did not address the topic of the review. After the screening, we arrived at 42 records assessed for eligibility, as we excluded 35 records that only had the title or abstract available, 21 were on dental implants, 10 about pulmonary disease, 9 were about chemical compounds, and 10 were solely about music theory. The entire exclusion process was performed by humans, not by automation tools. We added 11 studies that met the inclusion criteria identified via other methods (specialty books, organization, and a website). In the end, a total of 53 records met the inclusion criteria as can be seen in Figure 1.

### 3.1. Wind Instruments: Structure and Types of Contact with the Oral Cavity

#### Wind Instruments Are Composed of Two Main Parts

The tube, which serves as the body of the instrument, varies in size, shape, length, and materials depending on the specific instrument. It contains holes which can be closed directly with the fingers through keys or even with push-button pistons. The length of the tube can be adjusted by shaping a portion of it or by altering the position of the mouthpiece. This flexibility allows the musicians to mold the sound and achieve the desired musical expression. 

The mechanical mouthpiece is the component of the instrument that comes into contact with the musician’s mouth, serving as the intermediary between the tube and the oral cavity. The mouthpiece can be categorized as extra-oral or intra-oral. In the case of extra-oral mouthpieces, they are placed directly on the lips, such as the plate mouthpieces found on the transverse flutes, or the cup-shaped mouthpieces used with brass instruments. On the other hand, intra-oral mouthpieces are positioned inside the oral cavity, as seen with reed instruments. Reed instruments feature reed lamellae, which can be either single, such as those found on the saxophone or clarinet where the reed is attached to a mouthpiece, or double, such as the oboe where the two lamellas are held together directly on the instrument [1,7].

Regarding mouthpieces, the term can also refer to biophysiological mouthpieces, comprising all anatomic elements with the facial and oral cavity that contribute to the emission of musical sounds. In this context, the lips and cheeks play a role in achieving airtightness through contraction, while the tongue enables modulation of sound phrasing. The teeth provide support for the mouthpiece of the instruments, and supporting tissues help attenuate the forces exerted during instrumental practice, the temporomandibular joint facilitates adjusting the mandible’s position, and ultimately, the nervous system coordinates all of the elements [8].

### 3.2. Dental Classification of Wind Instruments Made by Strayer

The dental classification was established in 1939 by Strayer, an orthodontist and bassoonist. Strayer’s classification is based on the various adaptations of different mouthparts of the instruments, defining four classes [1,7]:

1. Class A: Basin mouthpiece instruments. This category includes all brass instruments, which consist of a cylindrical-conical tube that is coiled and features a piston or slide for adjusting the tube’s length. The pitch of the instrument is determined by the size of the tube, with larger tubes producing lower pitches. For instance, the tuba has a lower pitch compared to the cornet, and the helicon produces a lower pitch than the tuba. There are different types of mechanical mouthpieces, commonly known as basin or cup. These mouthpieces vary in size and shape with larger ones used for bass instruments, small rounded ones for trumpets, and thin narrow ones for flugelhorns. However, it is important to note that the characteristics of mouthpieces for the same instrument may differ.
2. Class B: This category also includes single reed woodwind instruments such as the clarinet and saxophone family. These instruments have a tapered mouthpiece that supports a single reed with a ligature. The size of the mouthpiece and the reeds varies depending on the specific instrument within the family; the sopranino is the smallest, followed by the soprano, alto, tenor, and finally the baritone. In the clarinet family, the E flat clarinet is the smallest, followed by the B flat clarinet, the C clarinet, alto clarinet, basset horn, and finally the bass clarinet. Furthermore, there are different size reeds available for each instrument. Musicians can choose between thinner or thicker reeds based on the desired effect.
3. Class C: Woodwind instruments with double reeds are classified in this category, including the families of the bassoon (bassoon and contrabassoon) and the oboe (oboe, oboe d’amore, and oboe baritone). The double reed consists of two reed blades connected by a wire wrapped around a brass tube with cork placed around it to facilitate insertion into the instrument. These instruments are played intra-orally, and the diameter of an oboe reed is approximately 0.5 mm. The space between the reed blades gradually narrows, resulting in a very thin opening.
4. Class D: instruments with a side mouthpiece or mouthpiece plate are classified in this category, including all transverse flutes: piccolo, flute, alto flute, and bass flute in C. The mouthpiece is positioned on the side of the instrument, requiring the musician to hold the flute parallel to their body. The mouthpiece is integrated into the instrument and consists of a plate that the musicians place their lower lip against. By controlling the tension of the lips, the flautist directs the airflow towards the flute’s embouchure hole, and the pitch increases as the lips are tightened [1,7].

### 3.3. The Impact of Wind Musical Instruments on the Oral Cavity

Since an increasing number of health professionals are encountering musicians, there is a growing concern regarding the effects of playing wind instruments on the stomatognathic system [9].

Dentists are frequently questioned about the potential negative impact of wind instrument playing on dental occlusion and other structures of the stomatognathic system, as it may significantly influence the alignment of the upper and lower anterior segments [10]. When performing on a wind instrument, several anatomical components of the oral cavity are involved, including the facial muscles, which regulate the airflow and seal the mouthpiece, the teeth that provide support, and the tongue, which contributes to phrasing. As different classes of instruments involve varying types of mouth contact, the consequences on the oral cavity also differ [11]. When examining the embouchure of wind instrumentalists, it is important to consider the specific dento-facial characteristics that may contribute to both anatomical and physiological changes [2]. Frequent playing can lead to musculoskeletal problems, particularly affecting the spine and upper extremities. Dental malpositions in wind instrument players are more frequently associated with a deep bite [3,12].

Generally, the effects of playing an instrument are continuously studied, as there is a tendency of parafunctional behavior in the cranio–cervical–mandibular complex. This behavior has minimal or no orthopedic impact on the morphology of the craniofacial region, but does affect the inclination of the lower frontal group [13]. It appears that a professional music career can be pursued without having an optimal jaw relationship [14] but this may result in later consequences that can be avoided or reduced by practicing physical exercises.

#### 3.3.1. Specific Influence of Class A Instruments

Class A instruments are positioned extra-orally, with the mouthpiece in perpendicular contact with the lips. In the case of the trumpet, the contact is distributed evenly between the upper and lower lip, while for the tuba and the trombone, the mouthpiece is placed predominantly on the upper lip (two thirds) and partially on the lower lip (one-third) [11]. The pressure generated during the trumpet playing is exerted backwards, towards the incisors. As the diameter of the mouthpiece increases, the pressure on the upper incisors becomes pronounced. The pressure exerted on the lips is around 500 g/m^2^, while the intra-oral air pressure can reach 25 kPa in higher register notes. This translates to a pressure of 190 mm of mercury for the internal air column, which is higher than the systolic arterial pressure [15]. This backward pressure can cause various pathologies, including constraints on the mandible and retro-discal pressure on the temporo-mandibular joint (TMJ). Therefore, maintaining proper posture is crucial in order to preserve postural balance [16]. Cervicofacial dystonias, especially in young musicians, have been observed, including dystonias affecting the canine muscle in trombone players [17]. We could also find lesions of the upper lips, such as the Satchmo syndrome experienced by Louis Armstrong (rupture of the orbicularis oris) [18,19]. In some cases, distension of the buccinator muscle due to excessive intra-oral pressure can be observed [18,20]. Bruxism may occur due to propulsion required during mouthpiece formation, while hypertonia of the orbicularis muscle can influence the antero-posterior position of the incisors. It appears that the tension of the lips has a greater impact on dental movements than the force exerted by the mouthpiece [21,22].

Playing brass instruments can affect the antero-posterior position of the incisors, particularly in combination with a delicate periodontium and consistent daily instrumental practice during the growth phase. This is especially relevant for young novice musicians who tend to exert greater oral pressure compared to experienced professionals who learn to reduce this pressure through increased facial muscle control. The pressure exerted on the maxillary incisors can reach the physiological limit of the tooth mobility within the alveolar socket. However, in adults with a healthy periodontium, no significant dental movements are observed [15,21,23]. Radiologically, rhizalysis of upper central incisors has been detected, along with enamel fractures and fissures [24] particularly when using a narrow mouthpiece [25]. However, these fractures typically occur as a result of accidental impact with the mouthpiece [26].

Due to wind instruments, patients may develop gingival pathologies. The pressure exerted by the mouthpiece, as well as the muscle activity, can lead to impaired capillary circulation of the periodontium, resulting in local stasis. This can cause marginal gingivitis, gingival recession, and gingival bleeding [24].

In terms of the soft palate, wind instrument playing can cause in velo-pharyngeal incompetence in trumpet players. This occurs when the soft palate herniates into the nasopharynx, leading to the passage of air through the nose during high-pressure instrumental practice, rendering playing the instrument impossible [27].

Furthermore, trumpet players may experience rhonchopathy, which is characterized by changes in the ligaments and muscle elasticity of the soft palate during playing, making the musician more prone to snoring [28].

#### 3.3.2. Specific Influence of Class B Instruments

Single-reed instrument mouthpieces are positioned intra-orally, with approximately half of the mouthpiece resting on the lower lip and inverted over the lower incisors. The upper incisors and the upper lip provide support. The beak of the mouthpiece exerts upward and forward forces on the upper incisors, as well as downward and backward forces on the lower incisors. The forces generated during playing the clarinet range from 2 to 5 kPam^2^, while for the saxophone they range from 2 to 8 kPam^2^ [29,30].

Class B instrument players may experience certain conditions including dental malposition in growing musicians. This often involves a vestibulo-position of upper incisors and a linguo-position of the lower incisors. Additionally, there may be a decrease in overbite and an increase in overjet. Oro-mandibular dystonia can also occur, as the musician struggles to stabilize the mandible during instrumental performance. This may result in excessive mouth opening and abnormal contact of the tongue, lips, and teeth with the mouthpiece [15,17].

TMJ joint dysfunctions can occur as a result of the retropulsion of the mandible when the mouthpiece beak is inserted [26]. In addition, adaptive hyperkeratosis, erosion, or ulceration may develop on the inner surface of the lower lip at the contact site with the lower incisors [17,25]. Dental and pulp pathologies can also arise, including chronic and acute pulpitis, which can lead to dental necrosis. These conditions may be attributed to micro-movements of the apex, causing inflammation of the vascular-nervous bundle, as well as micro-fractures of the enamel, facilitating the entry of ions into the endodontium [31]. Furthermore, abrasion of the incisal margins of the upper central incisors may occur [29].

#### 3.3.3. Specific Influence of Class C Instruments

The oboe, similar to the bassoon, features an intra-oral mouthpiece where the reed is positioned between the slightly elevated lips, resting on both the upper and lower incisors. However, the pressure exerted on the mouthpiece is primarily supported by the upper incisors. During oboe playing, forces are generated that direct upwards onto the upper incisors and downwards onto the lower incisors. We may also identify the following issues: pulpoliths with the pulp chamber of the maxillary central incisors and alveolysis, which can be influenced by factor such as the hardness of the reed, pressure applied to the mouthpiece, and frequency and duration of the instrument practice. Additionally, a reduced overjet and overbite may be observed [15,22].

#### 3.3.4. Specific Influence of Class D Instruments

The mouthpiece of class D instruments is extra-oral. The transverse flute is only in contact with the mouth at the level of the lower lip. Thus, the air pressures are less important than for the other classes of instrument where the forces are directed backwards for the upper incisors. Class D instrumentalists experience the following oral cavity influences: linguo-version of the mandibular incisors, increased overjet of the upper central incisors, TMJ dysfunctions due to recoil of the mandible during play, and creating pressure in the glenoidcavity [15,32].

### 3.4. Common Influence of Wind Instruments

Wind musical instruments, due to their close association with the oral cavity, share common characteristics that result in similar consequences in the oral sphere. These include:

The dental displacement: as previously mentioned, brass instruments exert a pressure of approximately 3 kg, woodwind instruments about 270 g, and flutes around 211 g. This pressure can lead to dental displacement. It is important to note that this differs from the forces applied in orthodontic treatment, which typically range from 35 to 60 g. Tartar formation: Playing wind instruments can cause increased salivary secretion that musicians often swallow at the end of their performance. This can lead to saliva stagnation in the oral cavity, resulting in tartar formation. Drying of the oral mucosa can occur as a result of mouth breathing or stress. Additionally, conditions such as intraductal stones or parotiditis may develop due to the retrograde progression of saliva in the Sténon’s duct, due to oral pressure during wind instrument playing [11].

Allergic dermatitis can occur on and around the mucous part of the lips. Some musicians develop an allergy to nickel in flutes and brass instruments, as well as to exotic woods in recorders and reeds in woodwind instruments [33].

Contact dermatitis resulting from friction and moisture at the mouthpiece has also been observed [20]. Herpes outbreaks can occur due to factors such as stress, fatigue, and lip trauma [14]. Irritativecheilitis can be caused by hypersalivation or excessive dryness [33].

Muscular fatigue, cramps, and tingling sensations in the orbicular oris, cheeks, and of the tongue have also been reported. Increased muscle activity during playing can lead to bone remodeling, including increased mandibular measurements, more developed dental arches, reduced facial length, and a smaller mandibular angle [34].

Due to significant airflow and respiratory system engagement, singers may be prone to developing respiratory conditions. Wind instruments, with their large inner surfaces, can harbor a large number of microorganisms that are not easily eliminated through standard cleaning methods. Consequently, the use of wind instruments can be a risk factor for respiratory tract infections. This risk is higher for immunosuppressed players, such as those undergoing immunosuppressive treatment, those living with AIDS, and diabetes. These players are more susceptible to opportunistic respiratory tract infections, especially tuberculosis [35].

Working in large collectives poses a risk for diseases transmitted through the fecal–oral route. Hepatitis A and Clostridium difficile infections are conditions that can be easily contracted due to their simple mode of transmission. Therefore, it is highly recommended for wind instrument players to prioritize sanitation, such as hand washing, safe water supply, and vaccination consideration, including the one against the hepatitis A virus [].

### 3.5. Analysis of Pathologies According to Each Kind of Wind Instruments

#### 3.5.1. Trumpets, Flugelhorns, Cornets

Trumpet players commonly experience labial muscle fatigue. In order to project air into the small and narrow mouthpiece positioned outside the oral cavity, the trumpet player must tightly contract their lips, leaving only a slit. This strong lip pressure necessary to prevent air leakage between the lips and the mouthpiece, can lead to muscular fatigue. Consequently, these musicians also complain of hypoesthesia of the lips, cheek fatigue, tingling in the parotid region, palato-version of the upper incisors, tingling of the lips, occasional mouth ulcers, and a decreased overjet [15,30,36].

#### 3.5.2. Trombones, Basses

These instruments have a larger and wider cup mouthpiece compared to the trumpet. As a result, the aperture that these musicians need to create with their lips is larger, requiring less muscle contraction. However, they still need to ensure a sealed passage of air between the oral cavity and the mouthpiece, which leads to similar pathologies. Additionally, due to the larger size of this instrument, a greater volume of air flow is required. This group of musicians commonly experience labial fatigue, hypoesthesia, tingling in the parotid region, palato-version of the upper incisors, occasional ulcers, tingling in the lips, dry mouth during playing, pain in the TMJ, laryngeal pain, and a sensation of galvanism [36,37].

#### 3.5.3. Clarinets

This instrument has an intra-oral mouthpiece. That requires the lips to surround the simple reed mouthpiece to ensure airtightness. The necessary muscle contractions can lead to muscle fatigue, occasional mouth ulcers, adaptive hyperkeratosis, cheilitis, increased overjet, lip ulceration, dry mouth, tingling in the parotid region, cheek fatigue, lingo-version of the lower incisors, and a lingual lesion. The impacts experienced by clarinet players are primarily of muscular and dermal nature [26,29].

#### 3.5.4. Saxophones

The saxophone group, similar to the clarinet, uses an intra-oral mouthpiece with a simple reed, around which the lips contract in order to maintain the air tightness. Prolonged practice can lead to labial muscle fatigue and hyperkeratosis of the inner surface of the lower lip. Saxophonists may also experience mouth dryness, lip ulceration, recurring tartar, occasional aphthous ulcers, increased overjet lingual fatigue, increased overjet, hypoesthesia of the lips, cheek fatigue, sensation of a lump in the throat and a diminished overbite, vestibulo-position of the upper central incisors, lingo-position of the lower incisors, and occasional TMJ pain. The pathologies observed in saxophonists primarily involve muscles and mucosal tissues [26,29].

#### 3.5.5. Oboe, Bassoon

These instruments feature a double reed intra-oral mouthpiece, requiring that the upper and lower lips be reversed around this double reed to create a sealed mouthpiece. As a result, the forces exerted by the lips are not countered by the direct forces of a mouthpiece and are instead directed backwards towards the teeth. Consequently, musicians often experience labial and cheek fatigue as well as a palato-version of the upper central incisors. Dry mouth following practice, diminished overbite, and diminished overjet are also common. The influences observed are primarily of a muscular and dental nature [15,30].

#### 3.5.6. Flutes

The mouthpiece is positioned laterally, outside the oral cavity. The musician rests their flute on their lower lip and directs the airflow using their upper lip. As a result, the mouthpiece does not form a tight seal, resulting in a somewhat muted sound compared to other instruments. The forces exerted on the teeth by the flute are also relatively weaker. Musicians may experience labial fatigue, dry mouth after extended periods of playing, herpetic outbreaks, tartar buildup, TMJ pain, retrognathia, increased overjet, slight tingling in the parotid, and occasional ulcerations [15,30].

### 3.6. The Beneficial Effects of Playing Wind Instruments in Children with Various Pathologies 

The fact that playing wind instruments can also have a beneficial effect on children from an oro-dental point of view is interesting. Thus, there are a number of anatomical peculiarities that can be corrected by playing these instruments. Of these, we note for all instruments, that the anatomical particularities that can be corrected by instrumental practice are velar incompetence, atony of the facial muscles, and lingual atony. For class A instruments, there is a good influence if there is a short upper lip, flabby lower lip, broad and extended tongue, superior alveolar prominence, or speech problems. Class B instruments could have a good influence on the upper retro-alveolus, class C could contribute to lip lengthening and infragnathia, and class D instruments have a good influence on a short and tense upper lip [38,39,40].

### 3.7. The Effect of Wind Instruments on the Face Muscles

The action of the facial muscles is essential for achieving the musical performance of wind instruments. 

Instrumentalists frequently experience facial muscle dysfunction, which has an impact on their professional performance and even leads to incapacitating symptoms.

Researchers have used electromyography (EMG) to examine facial muscle activity patterns involved in muscle stabilization, emission control, and articulation during musical activity under physiological conditions. In a study comparing the facial muscle activity of more experienced and less experienced clarinetists, it was found that the less experienced group exhibited higher overall facial muscle activity, indicating a potential risk of overuse. In contrast, the more experienced instrumentalists showed more optimized patterns of muscle activity [41].

Meanwhile, brass instruments appear to exert greater forces compared to woodwind instruments, with the trombone exerting the highest force, while the bassoon recorded the lowest force values [42].

Another concern among musicians is the impact on the musculoskeletal system when switching between instruments, which can lead to musculoskeletal stress and the risk of muscle pain and injury. Studies have been conducted on instrumentalists from large orchestras and theater groups, as well as those involved in studio recording sessions, to assess the level of facial muscle activation using EMG testing, depending on the instrument being played. Five that require significant facial muscle involvement were tested: bassoon, flute, oboe, saxophone, and clarinet [43].

**Figure 2:**
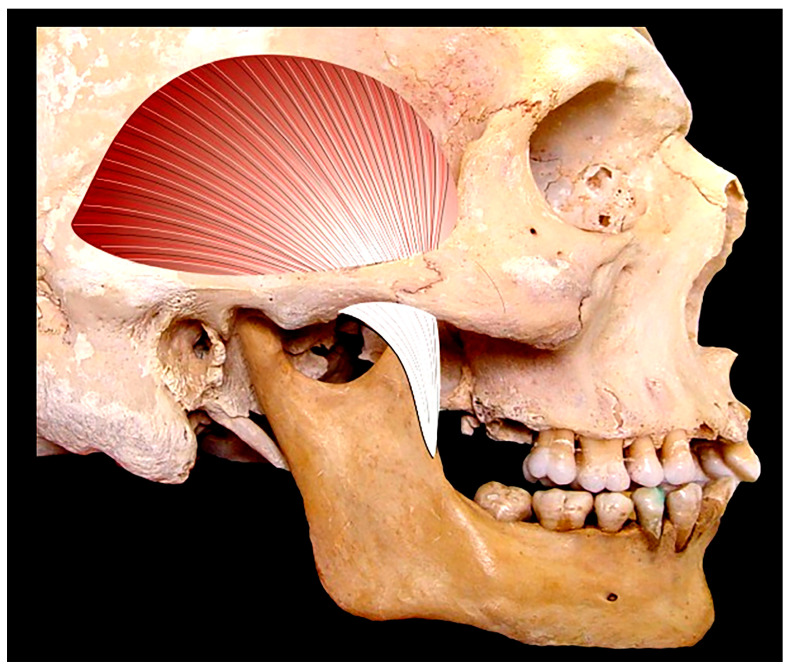
Temporalis muscle.

The muscles of the head (musculi capitis) are classified based on their anatomical and main action characteristics into masticatory muscles and cutaneous muscles or mimetic muscles. With the exception of the occipital muscle, all of these muscles are located on the anterior side, which is why they are also referred to as facial muscles. The masticatory muscles include the temporalis (Figure 2) and the masseter.

The masseter muscle is a powerful muscle located on the external side of the vertical branch of the mandible. It originates from the inferior edge of the zygomatic arch and the zygomatic bone (malar bone), and inserts at the angle of the mandible (gonion). When contracted, this muscle stands out prominently, contributing to a more angular appearance and a threating expression. Due to its volume and superficial position, it appears as the most prominent muscle during mastication in living individuals, although the temporalis muscle is stronger. Both work together in order to elevate the mandible, applying powerful force onto the upper jaw [44,45,46]. 

In the case of instrumentalists, the masseter muscle exhibited the most intense activity on the right side of the face. This pattern was consistent during the performance of each instrument, except the flute, where bilateral measurements were relatively equal. When comparing the instruments regarding masseter activation, it was found that the oboe had the highest activation patterns during musical activity, followed by the clarinet, saxophone, bassoon, and flute [43].

The temporal muscle (temporalis) is the most powerful masticatory muscle and is located in the temporal fossa. It inserts in a fan-like shape onto the temporal line of the frontal, parietal, and occipital bones, as well as on the deep side of the temporal fascia. Its fibers converge into a tendon and that passes under the zygomatic arch and inserts onto the coronoid process of the mandible [45].

When observing the temporal muscle, a noticeable difference in the level of activation between the bilateral muscles can be observed; the right temporal is much more engaged during each activity of each instrument. Among the instruments, the flute significantly activated the right temporal muscle more than any other woodwind instrument. The clarinet ranks second in terms of muscle activation during musical activity. Less temporal activity was found during musical performance of the saxophone, oboe, and bassoon [43].

According to an integrative review of ten studies, wind instruments frequently report clinical signs and symptoms of temporomandibular dysfunction [37].

Temporomandibular dysfunction is not the only condition that artists develop. Often, due to the prolonged effort required while playing for long periods of time, the nerve endings are stimulated, leading to pain in different areas. According to a study conducted in 2014, wind instrument artists most commonly experience pain in the temporomandibular joint, teeth, when closing the oral cavity, and pain in the neck [47].

In recent times, specific physical exercises have been developed to help alleviate pain in the neck area [48].

The muscles responsible for facial expression, known as facial muscles, have one end attached to the bones of the face and the other end attached to the deep side of the skin or they are situated within the thickness of the skin, such as the orbicularis.

**Figure 3:**
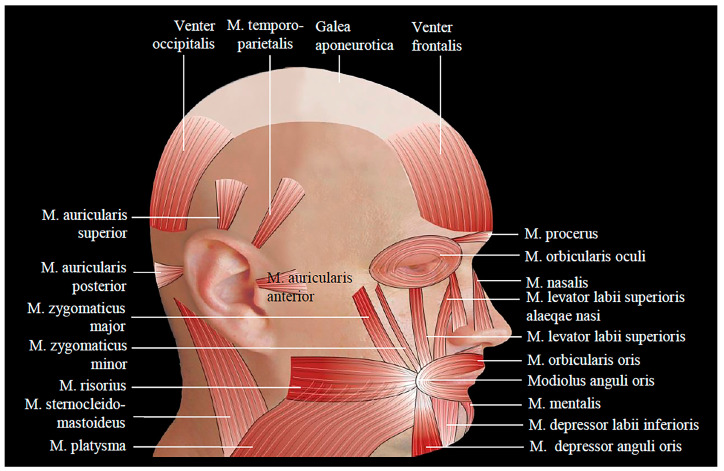
Muscles of facial expressions. Lateral view.

The zygomatic major (zygomaticus major) is located superficially in the central region of the cheek and is attached to the lateral side of the zygomatic bone. It descends downwards and medially towards the corner of the mouth, above the buccinators muscle. Its main function is to elevate and retract the corners of the mouth laterally. It is responsible for expressing laughter and gives the mouth a curved shape, concaving upwards [49] (Figure 3).

When comparing the left zygomaticus major muscle with the right one, significant differences in the activation patterns were observed depending on the instruments found, and used. The flute predominantly engages the left zygomaticus major muscle, compared to the right side. Furthermore, during musical activity on the flute, the left zygomaticus major muscle is more extensively engaged compared to the other instruments that also require its activation, such as the clarinet, oboe, bassoon, and saxophone [43].

The buccinator muscle is the widest and most powerful among the muscles of the mouth, positioned on a more profound plane compared to the others. It is located on the sides of the mouth within the cheek region. Posteriorly, it attaches to both jaws, and its fibers extend horizontally towards the commissures of the mouth. Upon contraction, it pulls them towards the lips, exerting pressure on the air and expelling it forcefully when whistling or when playing wind instruments. Due to this function, it is also referred to as the “trumpeters’ muscle”. The buccinator muscle contributes to both laughing and crying facial expressions, depending on its interaction with other muscles that act on the commissure of the lips [45].

This buccinator muscle is the primary muscle responsible for the structure and tightness of the cheek. When playing wind instruments, it contracts to expel air from the distended cheeks.

The fibers of the buccinator muscle converge towards the angle of the mouth, and fill the space between the upper and lower jaw. At the commissural node of the mouth, the fibers of the buccinator blend with other muscles, including the orbicularis oris, risorius, depressor angulioris, and zygomaticus major. Together they form a dense fibromuscular mass known as the modiolus [49].

In the anterior region of the neck, the two sternocleidomastoid muscles create a vertical triangle with the apex pointing downwards, located at the jugular notch of the sternum, and the base pointing upwards, along an imaginary horizontal line passing through the two angles of the mandible at the gonion points. This transverse line, together with the lower edge of the mandible, also forms a horizontal triangle. In the middle of this line, which serves as the common base of the two perpendicular triangles, lies the hyoid. The hyoid bone is a transverse bone situated within the thickness of the anterior neck muscles, and is not directly connected to the rest of the skeleton. The hyoid bone serves as the attachment point for the hyoid muscles, which shape the anterior region of the chin and neck within the space formed by the two triangles. Above the line, there are the suprahyoid muscles, and below it there are the subhyoid muscles. These muscles are responsible for elevating, lowering, or stabilizing the hyoid bone and lowering the mandible. They are the superficial muscles located in the mid-region of the neck. Vertically, they are divided by the infrahyoid white line and exist in symmetrical pairs: left and right [45].

The sternocleidomastoid muscle exhibits bilateral differences depending on the instrument played. The left sternocleidomastoid demonstrates greater muscle activity compared to the right side. Among the instruments, the bassoon, saxophone, and clarinet elicit significantly higher engagement of the sternocleidomastoid during performance compared to the flute and oboe [43].

These changes in facial muscles are a result of their repeated use and additional tension caused by increased intraoral pressure. In light of the SARS-CoV-2 pandemic, the need to prevent the spread of the virus through aerosols led to the development of studies in various fields, including the field of music. Thus, data were collected regarding levels in the upper airway, which can provide an insight into the additional tension experienced by facial mimicry muscles (especially the buccinator muscle and the orbicularis oculi). In wind instruments, the air is propelled up to a distance of 1.5 m from the player’s mouth. Considering that professional musicians produce sound by using the minimum amount of air necessary for a wide range of sounds, it is obvious that there is a significant increase in pressure within the lower and upper respiratory tracts. Thereby, the high pressure in the oral cavity, against which the buccinator and orbicularis oculi muscles must contract, can lead to muscle fiber hypertrophy and modifications in facial contours [50]. 

In athletes who overload certain muscle groups, it was found that serious postural deficiencies can occur, especially around the age of 11–14 years, due to the association of overload with a rapid growth of the bone system that is not associated and symmetrical with the development of soft tissues. Thus, special attention must be paid to the development of the facial and neck muscles in children and adolescents who frequently practice playing wind instruments.

### 3.8. Principles of Physical Therapy with Preventive and Curative Role in Pathologies Determined by Playing Wind Instruments

It is necessary to reduce pain in the oral cavity, facial, and neck muscles in order to improve the artistic performance of instrumentalists and prevent potential pathological conditions that may arise in this context.

We analyzed a set of breathing exercises in order to increase lung capacity and neck muscle toning exercise. These exercises can enhance the artistic performance of instrumentalists, by alleviating discomfort in the oral cavity, facial, and neck muscles, while also serving as a preventive measure against potential pathological conditions.

#### 3.8.1. Breathing Exercises

The ideal and beneficial way of breathing is through the diaphragm. When using the diaphragm, it is the lower abdomen that expands and contracts with each breath, rather than the chest. Diaphragmatic breathing enhances respiratory function and protects the lungs from environmental air pollution. Additionally, this type of breathing intensifies blood oxygenation in the lungs and, consequently, oxygenation of the cells.

Woodwind players are instructed to practice different types of breathing techniques to support this musical activity. There are different breathing exercises, such as humming breathing, alternate nostril breathing, compressed breathing, and many others that draw inspiration from Tai Chi [51].

Some types of exercises to increase lung capacity in wind instrument players are listed below:

1. Normal abdominal breathing—is the type of breathing practiced in Tai Chi and similar practices. It is performed slowly, deeply, with a deliberate engagement of the diaphragm. During inhalation, the abdomen expands, and during exhalation, it contracts. Abdominal breathing involves keeping the abdomen relaxed, which allows the diaphragm to descend easier, allowing air to enter the lower areas of the lungs. The movement of the diaphragm should contribute 75% of the respiratory capacity, while the intercostal muscles and the rib cage should contribute only 25%.
2. Prolonged breathing—characterized by prolonged and deep inhalation and exhalation. This type of breathing enhances the amount of oxygen intake and carbon dioxide elimination, provides energy to the peripheral cells during exhalation, and supplies significant energy to the bone marrow during inhalation.
3. Breathing exercise in supine position.
4. Breathing exercise in prone position.

Sitting in a chair, stretch your arms above your head and take a deep breath. Exhale slowly while bringing your arms down. Hold a smile on your face for three seconds. Practice this exercise for one minute.

5. Breathing exercise that mimics a yawn followed by a smile. This type of exercise is commonly used among instrumentalists, along with the following exercise.
6. Whistling breathing exercise. It is necessary to learn the fundamental breathing technique used by brass players, called diaphragmatic breathing.
  - Sit down on a chair and place your hands on the sides of your stomach.
  - With your lips closed, breathe gently through your nose and feel how your stomach rises/swells.
  - Once your lungs are full, keep your lips closed and exhale out slowly while humming, making a hissing sound, and lower your hands
  - Inhale through your nose again, then exhale as you hum.
  - Repeat for one minute [4].

#### 3.8.2. Exercises to Strengthen the Neck Muscles

The cervical region of the spine is a mobile and sensitive area of the musculoskeletal system. Typically, the instrumentalist adopts a specific body position in relation to the instrument, which can strain certain muscles of the neck when engaging them in an isometric exercise. Therefore, it is necessary to train all neck muscles through specialized exercises [4].

There are few types of exercises recommended by physical therapists that are particularly effective at this level.

Based on the movements that can be performed on this segment of the spine, we have selected the following exercises:

1. From a sitting position, bend the head forward as far as possible and hold for 5 s. Place one hand on the chin and press it towards the chest, while the other hand applies pressure on the back of the head. Hold for 5 s.
2. From a sitting position, tilt the head backward as far as comfortable and hold for 6 s. To improve the effectiveness of the exercise, push the chin and the forehead backwards to achieve a wider extension. It is maintained for 6 s.
3. From a sitting position, tilt the head to the left side as far as possible and hold for 6 s. Then force this position by pushing the head with the hand. Hold for 6 s.
4. Repeat the same exercise as in step 3, but this time tilt the head to the right side.
5. Rotational movements of the head are beneficial. Perform slow and wide rotation in a rhythmic manner. These movements can also be used as relaxation exercises.

These isometric neck exercises can be varied based on the basic movements we have presented.

Breathing rhythm is an important aspect: perform the exercise during inhalation, and return to the initial position during exhalation.

It is recommended to practice these types of exercises 4–5 times a week, in order to tone the neck muscles [5,52].

It is also necessary to discuss the practice of these exercises with a doctor when they engage a sensitive area such as the cervical spine.

#### 3.8.3. Exercises for the Oro-Facial Muscles, Mandible Mobilizing Muscles, and the TMJ

Maintaining dento-facial balance in wind instrumentalists is a complex subject that requires a multipolar approach ranging from exercises to tone the oro-facial and mandible mobilizing muscles, TMJ relaxation, maintaining proper posture, and ensuring optimal oral health. Here are some examples of facial muscle exercises recommended for wind instrumentalists:

1. The most common exercise, which is performed before each rehearsal, for wind instrumentalists is vocalization (a, e, i, o, u) and pronunciation of words/facial expressions that involve different mouth shapes, but exaggerated to the maximum: wide open horizontally, wide open vertically, and closed with puckering of the lip. Perform five repetitions of each exercise, slowly, holding the vowel/word/expression for 5 s, then 10 repetitions of each at a medium speed, then at a high speed.
2. From a relaxed seated position on the sofa, in cervical extension, push the lips as much as possible and pucker them, holding for 10 s, then extend them into a very wide smile, holding for 10 s. Repeat daily, in three sets of 10 repetitions each.
3. The movement of “sucking in” the cheeks, alternating with “puffing out” the cheeks. Hold each expression for 8–10 s. Repeat 10 times daily.
4. Chewing gum for 5 min on each side of the mouth, left–right.
5. Blowing up balloons–this exercise affects the oro-facial muscles and develops respiratory capacity.
6. Puffing out the cheeks–take a deep breath and, on exhalation, puff out the cheeks as much as possible. Hold the air in the cheeks for 5–10 s, being aware of the muscle tension without it being painful. Slowly release the air and repeat the exercise 5–10 times. This exercise helps tone the fibers of the orbicularis oris and buccinator muscles, which are involved in controlling the airflow during wind instrument playing.
7. Variation of the cheek puffing exercise–take a deep breath and, on exhalation, puff out the cheeks as much as possible. Press the palms against the cheeks, trying to deflate them, but at the same time, resist the deflation for a few seconds until they can be successfully deflated. Repeat the exercise 5–10 times.
8. Lip buzzing/rolling—with the lips in contact, inhale air into the chest and exhale the air through the lips, causing them to vibrate (the effect is a sound similar to a “BRR” made by babies). Start slowly, then increase the speed of exhalation while maintaining control of the lips to achieve vibration and forceful airflow. Repeat 3 to 5 times.
9. Tongue strengthening—position the tip of the tongue against the palate in the anterior third behind the front teeth without dental contact. Push the tongue firmly against the palate, holding for a few seconds, then relax the tongue and repeat the movement. Perform the exercise five times, ensuring that the support is always bony and not dental.
10. Tongue toning and mobility improvement—trace circles with the tip of the tongue by moving it from one oral commissure to the other, within the mouth, five times in one direction, then five times in reverse. Next, the same type of movement is performed from one oral commissure to the other, but describing the circle with the tip of the tongue outside the mouth. These exercises aim to strengthen the tongue muscles, which is important for controlling it while playing an instrument, and to counteract the pressure exerted by the instrument on the teeth.
11. Opening and closing the mouth–slowly and gently open your mouth as wide as possible without causing pain or discomfort. Hold the open position for a few seconds, then slowly close your mouth. Repeat this movement several times, focusing on achieving a smooth and fluid motion of mouth opening. This exercise helps promote mobility and flexibility of the temporomandibular joint, relieving tension in that area.
12. In cases of occurrence of joint noises at the TMJ, the following exercise can be practiced, which is aimed at strengthening the ligaments of the TMJ and relaxing the mobilizing muscles of the mandible [53]. While maintaining an upright posture, close the mouth until the teeth lightly touch. Avoid clenching the teeth. Place the tip of the tongue on the palate, just behind the upper incisors. Move the tip of the tongue backward towards the soft palate at the back of the mouth, as far as it can comfortably go, while keeping the teeth lightly in contact. Maintain the tongue in this posterior position and slowly open the mouth until the tongue feels tension. Stop the mouth opening and maintain this position for five seconds, then close the mouth in a relaxed manner. Repeat this exercise for 5 min daily. In the beginning, it is recommended to perform the exercise in front of a mirror to control and visualize the vertical mouth opening movement. The movement should not be deviated laterally. Gradually increase the duration, starting with 5 min twice a day in the first week, then increasing the frequency to more than twice a day from the second week onwards. Improvements in the TMJ status should occur after 2–3 weeks of exercises. Repeat as necessary.

Practicing specific exercises for the oro-facial and the mandible mobilizing muscles involved in playing wind instruments can indirectly prevent dental changes and TMJ dysfunctions. These exercises can help achieve optimal muscle tone, counteracting the pressure exerted by the instrument on the teeth and the entire stomatognathic system.

## 4. Conclusions

1. The pathologies were analyzed according to each type of wind instrument: trumpets, cornets, trombones, bass, clarinets, saxophones, oboes, bassoons, and flutes. Brass instruments were found to exercise greater forces compared to wooden ones.

2. Moreover, it was observed that lip tension has a greater impact on tooth movement than mouthpiece force.

3. Regarding the influence of musical instruments on facial muscles, the data indicate that less experienced instrumentalists exhibit higher average facial muscle activity, which may lead to overuse, whereas more experienced instrumentalists demonstrate more optimized muscle activity patterns.

4. Wind instruments often report clinical signs and symptoms of temporomandibular dysfunction. These changes in the facial muscles are the result of repetitive use and the additional tension caused by increased intra-oral pressure. The high pressure in the oral cavity leads to hypertrophy of the muscle fibers, particularly in the facial regions.

5. Breathing and neck muscle toning exercises are valuable in preventing the anatomical and functional changes associated with wind instrument playing. Furthermore, these exercises help alleviate pain in the oral cavity, in the facial muscles, and neck, while also serving as preventive measures against the development of pathological conditions in this context.

6. The implementation of targeted exercises designed to activate the oro-facial and mandible mobilizing muscles implicated in the performance of wind instruments, has the potential to facilitate the attainment of an ideal muscular state, effectively counterbalancing the forces exerted by the instrument on both the dentition and the stomatognathic system, mitigating and preventing dental malposition and TMJ dysfunctions in an indirect manner.

7. The novelty of this article lies in its interdisciplinary approach to addressing these phenomena, encompassing both prevention and correction aspects.

## Data Availability

The data that support the findings of this study are available from the corresponding author.
