# Correction: Macovei et al. Changes in Dento-Facial Morphology Induced by Wind Instruments, in Professional Musicians and Physical Exercises That Can Prevent or Improve Them—A Systematic Review. *Life* 2023, *13*, 1528

**DOI:** 10.3390/life15050761

**Published:** 2025-05-09

**Authors:** Georgiana Macovei, Raluca Minea, Iarina Teodora Dumitraș, Cosmin Andrei Precup, Liliana Baroiu, Alexandru Nechifor, Adina Oana Armencia, Ana Cristina Lese

**Affiliations:** 1 Department of Oral and Dental Diagnostics, “Gr. T. Popa” University of Medicine and Pharmacy Iaşi, No. 16, Universității Street, 700115 Iași, Romania; dr_geo_m@yahoo.com (G.M.); iarina.dt@gmail.com (I.T.D.); oanaarmencia@yahoo.com (A.O.A.); 2 Department of Art History, Artistic Anatomy, Faculty of Visual Arts and Design, “George Enescu” National University of the Arts Iaşi, No. 189, Sărărie Street, 700451 Iaşi, Romania; 3 Department of Balneology, Medical Recovery and Rheumatology, “V.Babeş”University of Medicine and Pharmacy Timişoara, No. 2, Piața EftimieMurgu Street, 300041 Timișoara, Romania; precup.cosmin2@gmail.com; 4 Clinical Medical Department, Faculty of Medicine and Pharmacy,” Dunărea de Jos” University of Galați, No. 47, Domneasca Street, 800008 Galaţi, Romania or liliana.baroiu@ugal.ro (L.B.); alexandrunechiformed@yahoo.com (A.N.); 5 Infectious Diseases Department, ”Sf. Cuv. Parascheva” Clinical Hospital of Infectious Diseases Galaţi, No. 393, Traian Street, 800179 Galaţi, Romania; 6 Multidisciplinary Integrated Center of Dermatological Interface Research Center (MICDIR), ”Dunărea de Jos” University of Galați, No. 47, Domneasca Street, 800008 Galaţi, Romania; 7 Department of Design, Faculty of Visual Arts and Design, “George Enescu” National University of the Arts Iaşi, No. 189, Sărărie Street, 700451 Iaşi, Romania; analese2000@yahoo.com

Following publication, concerns were raised regarding the relevance of references [36,37,53] in this publication [1]. The Editorial Board conducted an investigation, which determined that these references are irrelevant to the content of the article. As a result, following discussion with the authors, the Editorial Board has decided to remove the irrelevant citations from this publication [1].

With this correction, the order of some references has been adjusted accordingly. The authors state that the scientific conclusions are unaffected. This correction was approved by the Academic Editor. The original publication has also been updated.

[36] Cambrea, S.C.; Arghir, O.C.; Rascu, A.; Petcu, C.L. Biochemical features of an acute viral hepatitis A outbreak. *Rev. Chim.* **2018**, *69*, 1447–1450.

[37] Baroiu, L.; Beznea, A.; Please Condratovici, C.; Onisor, C.; Grigore, C.A.; Topor, G.; Rugină, S. Comparative Effectiveness of Vancomycin and Metronidazole for the Initial Episode of Nonsevere Clostridium Difficile Infection. *Rev. Chim.* **2019**, *70*, 3741–3745.

[53] Iordan, D.A.; Mocanu, G.D.; Mocanu, M.D.; Munteanu, C.; Constantin, G.B.; Ilie, O.N.U.; Nechifor, A. Age-Related, Sport-Specific Dysfunctions of the Shoulder and Pelvic Girdle in Athletes Table Tennis Players. Observational Study. *Balneo Res. J.* **2021**, *12*, 337–444.
